# Association Between Early Breast Milk Feeding Proportion and Discharge Weight z-Score in Preterm Infants

**DOI:** 10.3390/children13040498

**Published:** 2026-04-01

**Authors:** Chang Mi Kwon, Ji Eun Jeong, Eun Ah Kim, So Hee Lee, Sang Gyu Kwak

**Affiliations:** 1 Department of Pediatrics, Daegu Catholic University Medical Center, Daegu Catholic University Hospital, Daegu 42472, Republic of Korea; cmk1121@dcmc.co.kr; 2 Department of Pediatrics, Daegu Catholic University Medical Center, Daegu Catholic University School of Medicine, Daegu 42472, Republic of Korea; neojeong@cu.ac.kr (J.E.J.); eunahkim@cu.ac.kr (E.A.K.); sohee0213@cu.ac.kr (S.H.L.); 3 Department of Medical Statistics, Daegu Catholic University School of Medicine, Daegu 42472, Republic of Korea

**Keywords:** breast feeding, growth, human milk, neonatal intensive care unit, preterm infants, restricted cubic spline

## Abstract

**Highlights:**

**What are the main findings?**

- A higher proportion of breast milk feeding during the first 14 days of life was associated with a higher discharge weight z-score in baseline-adjusted analyses.
- The dose–response relationship was approximately linear, and exploratory mediation analysis suggested that late-onset sepsis may partly mediate this association.

**What are the implications of the main findings?**

- Modeling breast milk exposure as a continuous proportion may provide more informative evidence than categorical feeding classifications.
- The observed association between early breast milk exposure and growth in preterm infants may partly reflect pathways involving reduced infectious morbidity.
- These findings should be interpreted cautiously and should not be considered evidence of a causal relationship, given the substantial potential for residual confounding by prematurity and illness severity.

**Abstract:**

Background/Objectives: Breast milk is recommended as primary enteral nutrition for preterm infants, but the quantitative association between early breast milk feeding proportion and short-term growth remains unclear. We examined the relationship between early breast milk feeding proportion and discharge weight z-score in preterm infants. Methods: This single-center retrospective cohort study included preterm infants admitted to a neonatal intensive care unit between January 2024 and December 2025. Early breast milk feeding proportion was defined as the percentage of breast milk intake among total enteral nutrition during the first 14 days of life. The primary outcome was discharge weight z-score based on the Fenton growth reference. Linear regression, restricted cubic spline analysis, and exploratory mediation analysis were performed. Results: Among 1174 preterm infants, a higher early breast milk feeding proportion was independently associated with a higher discharge weight z-score in the primary multivariable model adjusted for gestational age, sex, and initial mechanical ventilation. A 10% increase in breast milk feeding proportion was associated with an increase of 0.18 in discharge weight z-score (β = 0.18; 95% CI, 0.09–0.26; *p* < 0.001). Restricted cubic spline analysis showed an approximately linear association. In sensitivity analyses additionally adjusted for late-onset sepsis and necrotizing enterocolitis, the association was no longer statistically significant. Exploratory mediation analysis suggested that the association may be partly explained through pathways involving late-onset sepsis, whereas the mediating role of necrotizing enterocolitis appeared to be more limited. Conclusions: In baseline-adjusted analyses, a higher early breast milk feeding proportion was associated with a higher discharge weight z-score; however, this association was attenuated and no longer statistically significant after additional adjustment for major neonatal complications. These findings should be interpreted cautiously and should not be considered evidence of a causal relationship, given the substantial potential for residual confounding by prematurity and illness severity.

## 1. Introduction

Breast milk is widely recognized as the optimal source of nutrition for infants, providing substantial short- and long-term benefits for growth, immune function, and neurodevelopment [1]. In preterm infants, particularly those admitted to the neonatal intensive care unit (NICU), breast milk feeding has been associated with reduced risks of infection, necrotizing enterocolitis (NEC), and mortality, while supporting overall clinical outcomes [2,3,4,5]. Despite advances in neonatal care, postnatal growth failure remains a persistent challenge among preterm infants. Extrauterine growth restriction continues to be frequently observed during NICU hospitalization and has been linked to adverse neurodevelopmental outcomes later in life [4,6,7]. Adequate early nutritional support is therefore a critical component of neonatal management, and international guidelines emphasize the importance of enteral nutrition, preferably with human milk, to promote optimal growth in this vulnerable population [6].

Previous studies have demonstrated that early exposure to human milk is associated with improved clinical outcomes in very low birth weight infants, including lower rates of late-onset sepsis and NEC [5,8]. More recent cohort studies have also highlighted factors influencing breast milk provision during NICU hospitalization, underscoring the clinical relevance of breast milk feeding practices in contemporary neonatal care settings [9]. However, much of the existing literature has focused on binary comparisons (e.g., human milk versus formula) or categorical definitions of feeding exposure, which may not fully capture the potential dose–response relationship between the proportion of breast milk intake and growth outcomes.

From a methodological perspective, categorizing continuous exposure variables can lead to loss of information and reduced statistical power. Dose–response analyses using flexible modeling approaches, such as restricted cubic splines, allow for a more nuanced assessment of continuous exposures and can reveal potential non-linear associations that might otherwise be overlooked [10,11]. Although such methods have been increasingly applied in public health research, their use remains limited in neonatal nutrition studies. Furthermore, growth outcomes in preterm infants are commonly evaluated using standardized growth references. The Fenton growth chart provides a widely accepted framework for assessing weight-for-age z-scores in preterm populations and allows for meaningful comparison of growth trajectories across studies [12].

However, the association between human milk feeding and postnatal growth in preterm infants remains controversial. While human milk is associated with important clinical benefits, several studies have reported slower early weight gain with higher proportions of human milk intake in very low birth weight infants, including dose–response relationships [13,14]. In contrast, systematic reviews have shown that findings remain inconsistent across studies [15].

These inconsistencies may be explained by differences in study populations, nutritional practices such as fortification, and the analytical handling of feeding exposure, which has often been categorized rather than analyzed as a continuous variable. Therefore, the quantitative dose–response relationship between early human milk exposure and growth outcomes remains insufficiently characterized.

In this context, the present study aimed to examine the association between early breast milk feeding proportion and discharge weight z-score in preterm infants admitted to the NICU. By modeling breast milk feeding proportion as a continuous exposure and applying restricted cubic spline analysis, we sought to evaluate both the magnitude and potential nonlinearity of this association, while adjusting for relevant clinical covariates. This approach may provide a more refined understanding of the relationship between early breast milk exposure and growth outcomes in preterm infants.

## 2. Materials and Methods

### 2.1. Study Design and Population

This study was a single-center, retrospective observational cohort study conducted using electronic medical record (EMR) data from preterm infants admitted to the neonatal intensive care unit (NICU) at Daegu Catholic University Medical Center. Consecutive preterm infants who were admitted to the NICU between 1 January 2024, and 31 December 2025 were screened for eligibility.

Infants were eligible for inclusion if they were born at a gestational age of <32 weeks or with a birth weight of <1500 g, were admitted to the NICU within 24 h after birth, and received enteral nutrition during hospitalization with available records of breast milk and formula intake during the early postnatal period. Availability of body weight measurements at discharge was required to assess the primary outcome. Infants were excluded if they had major congenital anomalies or chromosomal abnormalities, gastrointestinal malformations that precluded standard enteral feeding (e.g., intestinal obstruction or atresia), died within 72 h after birth, or had substantial missing data in either the exposure variable (early breast milk feeding proportion) or the primary outcome (discharge weight). Cases with incomplete or ambiguous documentation of early enteral feeding were also excluded.

After application of the inclusion and exclusion criteria, a total of 1174 preterm infants were included in the final analytic cohort. The association between early breast milk feeding proportion during the first 14 days of life and discharge weight z-score was subsequently evaluated.

The study protocol was reviewed and approved by the Institutional Review Board of Daegu Catholic University Medical Center (IRB No. DCUMC 2026-01-056). The requirement for informed consent was waived owing to the retrospective nature of the study and the use of de-identified patient data. All study procedures were conducted in accordance with the Declaration of Helsinki and relevant institutional guidelines.

### 2.2. Data Collection and Variables

Clinical data were extracted retrospectively from the institutional clinical data warehouse and electronic medical records of Daegu Catholic University Medical Center. All data were de-identified prior to analysis. Variables collected for this study included perinatal characteristics, nutritional variables, and clinical outcomes during NICU hospitalization.

#### 2.2.1. Exposure Variable

The primary exposure variable was the early breast milk feeding proportion, defined as the percentage of breast milk intake relative to the total enteral nutrition volume during the first 14 days of life. Breast milk intake included both mother’s own milk and donor human milk, whereas formula intake comprised commercially available preterm formula. These two sources of human milk were recorded collectively in the database and could not be distinguished separately in the present analysis.

The breast milk feeding proportion was calculated as follows:

(1) $$\mathrm{Breast}\;\mathrm{milk}\;\mathrm{proportion}\;(\%)=\frac{\mathrm{Total}\;\mathrm{breast}\;\mathrm{milk}\;\mathrm{volume}}{\mathrm{Total}\;\mathrm{enteral}\;\mathrm{feeding}\;\mathrm{volume}}\times 100$$

The exposure variable was analyzed both as a continuous variable and as a categorical variable in descriptive analyses, with infants classified into high and low breast milk feeding groups based on the median value.

#### 2.2.2. Outcome Variables and Covariate Variables

The primary outcome was discharge weight z-score, calculated using the Fenton growth reference for preterm infants. Body weight at discharge was standardized according to sex and postmenstrual age to derive the corresponding z-score. Postmenstrual age was calculated as the sum of gestational age at birth and postnatal age at discharge.

Covariates were selected a priori based on clinical relevance and prior literature. Perinatal variables included gestational age (weeks), sex, birth weight, mode of delivery, multiple birth status, and exposure to antenatal corticosteroids. Clinical variables during NICU hospitalization included initial mechanical ventilation, surfactant administration, late-onset sepsis, necrotizing enterocolitis, and length of hospital stay. Gestational age was recorded in completed weeks, and birth weight was measured in grams. Late-onset sepsis was defined as culture-proven or clinically diagnosed sepsis occurring after 72 h of life. Necrotizing enterocolitis was defined according to Bell stage II or higher based on standard clinical and radiologic criteria.

#### 2.2.3. Nutritional Management and Clinical Care

During the study period, preterm infants were managed according to a standardized nutritional support protocol in the NICU. Parenteral nutrition was initiated shortly after birth and consisted of amino acids, glucose, and intravenous lipid emulsions, with gradual advancement according to routine clinical practice and the infant’s metabolic tolerance.

Enteral feeding was initiated when clinically feasible, typically using mother’s own milk as the first choice. Feeding advancement was performed gradually based on feeding tolerance, including assessment of gastric residuals, abdominal examination findings, stool patterns, and overall clinical stability.

Donor human milk was used when mother’s own milk was unavailable or insufficient, particularly in preterm infants considered at higher risk for feeding intolerance or necrotizing enterocolitis. Preterm formula was administered when human milk (maternal or donor) was unavailable or when additional supplementation was deemed necessary by the attending neonatologist.

Human milk fortifier was introduced when enteral feeding reached the institutional threshold of 100 mL/kg/day, according to standard NICU practice, to ensure adequate nutrient intake for growth.

Because of the retrospective nature of the study, there may have been minor variations in the timing and progression of feeding and nutritional support depending on the infant’s clinical condition and physician judgment.

Discharge was considered when infants achieved clinical stability, including the ability to maintain body temperature in an open crib, tolerance of full enteral feeding, consistent weight gain, and no requirement for acute inpatient neonatal care.

### 2.3. Statistical Analysis

Continuous variables were summarized as means with standard deviations, and categorical variables were presented as frequencies and percentages. Baseline characteristics were compared between high and low breast milk feeding proportion groups, defined by the median value, using the independent two-sample *t*-test or Mann–Whitney U test for continuous variables and the chi-square test or Fisher’s exact test for categorical variables.

For univariate analysis, simple linear regression analyses were first performed to examine the association between each candidate variable and discharge weight z-score. Regression coefficients (β) with 95% confidence intervals (CIs) were reported. For multivariate analysis, multiple linear regression models were then constructed to evaluate the independent association between early breast milk feeding proportion and discharge weight z-score. Birth weight was not included in the primary model due to strong collinearity with gestational age and conceptual overlap with baseline maturity. Multicollinearity among covariates was assessed using variance inflation factors (VIFs), and all variables included in the final model had VIF values < 5, indicating no significant multicollinearity. The primary model was adjusted for gestational age, sex, and initial mechanical ventilation based on clinical relevance. A sensitivity analysis was conducted with additional adjustment for late-onset sepsis and necrotizing enterocolitis to assess the robustness of the association. Regression results were presented as β coefficients with 95% confidence intervals. For interpretability, regression coefficients are presented per 10% increase in breast milk feeding proportion, although the variable was modeled continuously on its original percentage scale.

To explore potential non-linear relationships between early breast milk feeding proportion and discharge weight z-score, a restricted cubic spline model was fitted. Knots were placed at the 35th and 65th percentiles of the breast milk feeding proportion, with boundary knots set at the minimum and maximum observed values. The spline model was adjusted for gestational age, sex, and initial mechanical ventilation. The presence of nonlinearity was assessed visually and by comparing model fit. Adjusted marginal effects of early breast milk feeding proportion on discharge weight z-score were estimated from the multivariable regression model and plotted relative to the median breast milk feeding proportion to facilitate clinical interpretation.

To further explore the role of postnatal complications in the association between early breast milk feeding proportion and growth outcomes, an exploratory mediation analysis was performed. Separate mediation models were constructed for late-onset sepsis and necrotizing enterocolitis. The mediator was modeled using logistic regression, and the outcome (discharge weight z-score) was modeled using linear regression. Average causal mediation effects (ACME), average direct effects (ADE), and total effects were estimated using bootstrap methods.

Cases with missing data in the exposure or primary outcome variables were excluded from the analysis (complete-case analysis). No imputation methods were applied. The extent of missing data for other covariates was minimal and did not materially affect the analytic sample. All statistical analyses were performed using Python (version 3.11) with the ‘statsmodels’ and ‘patsy’ packages. A two-sided *p* value < 0.05 was considered statistically significant.

## 3. Results

### 3.1. Baseline Characteristics

A total of 1174 preterm infants were included in the final analysis and were divided into high and low breast milk feeding proportion groups according to the median value of breast milk intake during the first 14 days of life (Table 1). Infants in the high breast milk group had a significantly higher gestational age at birth compared with those in the low breast milk group (29.66 ± 1.60 vs. 28.25 ± 1.89 weeks, *p* < 0.001) and higher birth weight (1219.17 ± 204.90 vs. 1092.26 ± 254.60 g, *p* < 0.001). The proportion of male infants did not differ significantly between groups. Clinical characteristics also differed between groups. The high breast milk group had lower rates of initial mechanical ventilation and surfactant use than the low breast milk group, as well as a lower rate of late-onset sepsis (11.2% vs. 26.1%, *p* < 0.001). Length of hospital stay was significantly shorter in the high breast milk group (47.36 ± 19.37 vs. 60.83 ± 21.88 days, *p* < 0.001). Discharge weight did not differ significantly between groups.

### 3.2. Univariate Analysis

In univariable linear regression analyses, a higher early breast milk feeding proportion was significantly associated with a higher discharge weight z-score (Table 2). A 10% increase in breast milk feeding proportion was associated with an increase of 0.24 in discharge weight z-score (β = 0.24; 95% CI, 0.16–0.32; *p* < 0.001). Gestational age and birth weight were positively associated with discharge weight z-score, whereas male sex, surfactant use, late-onset sepsis, and necrotizing enterocolitis were negatively associated with the outcome. Multiple birth status and antenatal steroid exposure were not significantly associated with discharge weight z-score.

### 3.3. Multivariate Analysis

In the primary multivariable linear regression model adjusted for gestational age, sex, and initial mechanical ventilation, early breast milk feeding proportion remained independently associated with discharge weight z-score (Table 3). A 10% increase in breast milk feeding proportion was associated with an increase of 0.18 in discharge weight z-score (β = 0.18; 95% CI, 0.09–0.26; *p* < 0.001). Male sex and initial mechanical ventilation were independently associated with lower discharge weight z-scores, whereas gestational age showed a borderline association. 

In the sensitivity analysis additionally adjusted for late-onset sepsis and necrotizing enterocolitis, the association between early breast milk feeding proportion and discharge weight z-score was no longer statistically significant (β = 0.005; 95% CI, −0.002–0.013; *p* = 0.171). Late-onset sepsis and necrotizing enterocolitis were both strongly associated with lower discharge weight z-scores.

### 3.4. Dose–Response Relationship and Marginal Effects

**Figure 1:**
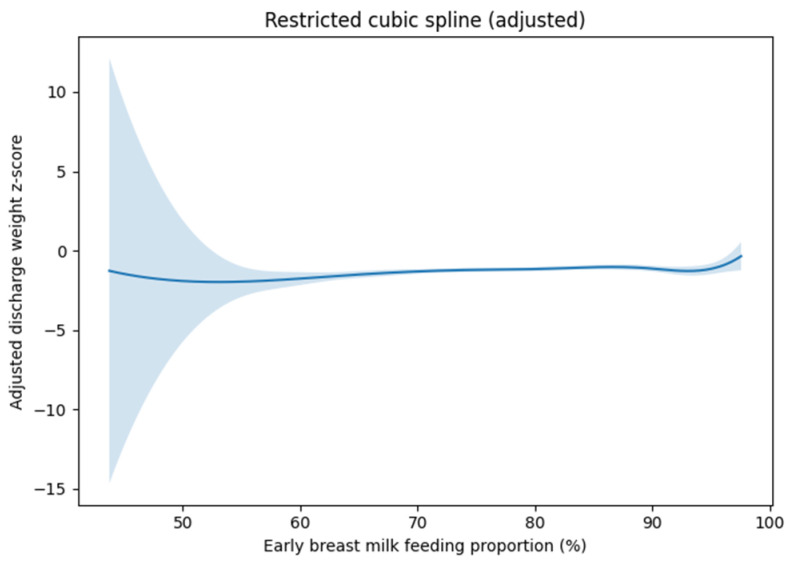
Restricted cubic spline illustrating the association between early breast milk feeding proportion and discharge weight z-score. The model was adjusted for gestational age, sex, and initial mechanical ventilation. The solid line represents the adjusted mean estimate, and the shaded area indicates the 95% confidence interval. The spline demonstrates an approximately linear association across the observed range, with wider confidence intervals at the extremes reflecting fewer observations.

**Figure 2:**
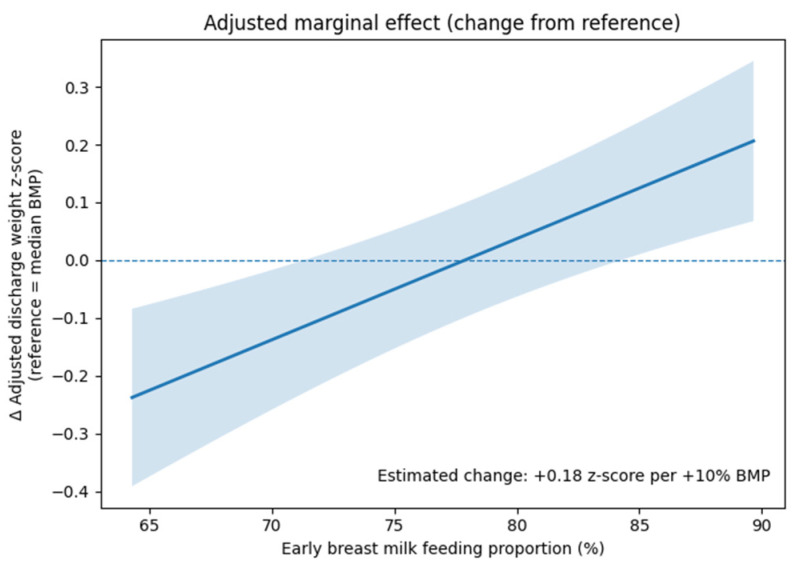
Adjusted marginal effect of early breast milk feeding proportion on discharge weight z-score. The figure depicts the adjusted change in discharge weight z-score relative to the median early breast milk feeding proportion, derived from a multivariable linear regression model. The solid line indicates the point estimate, and the shaded area represents the 95% confidence interval. The model was adjusted for gestational age, sex, and initial mechanical ventilation. An increase of 10% in early breast milk feeding proportion was associated with an estimated increase of 0.18 in discharge weight z-score.

Restricted cubic spline analysis was performed to explore potential non-linear associations between early breast milk feeding proportion and discharge weight z-score (Figure 1). The restricted cubic spline analysis suggested an approximately linear association within the observed range of the cohort. However, this relationship should not be interpreted as supporting extrapolation to extreme hypothetical values (e.g., 0% to 100%). Confidence intervals widened at the extremes, reflecting fewer observations. The adjusted marginal effect plot demonstrated a gradual increase in discharge weight z-score with increasing early breast milk feeding proportion relative to the median value (Figure 2). An increase of 10% in early breast milk feeding proportion was associated with an estimated increase of approximately 0.18 in discharge weight z-score in the primary adjusted model.

### 3.5. Mediation Analysis

An exploratory mediation analysis was conducted to assess whether late-onset sepsis and necrotizing enterocolitis mediate the association between early breast milk feeding proportion and discharge weight z-score. For late-onset sepsis, a statistically significant indirect effect was observed (ACME = 0.104; 95% CI, 0.071–0.143; *p* < 0.001), with an estimated proportion mediated of approximately 56%. The direct effect did not reach statistical significance (ADE = 0.081; 95% CI, −0.003 to 0.165; *p* = 0.059).

In contrast, for necrotizing enterocolitis, the indirect effect was smaller and did not reach statistical significance (ACME = 0.031; 95% CI, −0.000 to 0.066; *p* = 0.053), while the direct effect remained statistically significant (ADE = 0.144; 95% CI, 0.064–0.229; *p* < 0.001). These findings suggest that the association between breast milk feeding and growth may be partly mediated through late-onset sepsis, whereas the mediating role of necrotizing enterocolitis appears to be more limited.

## 4. Discussion

In this retrospective cohort study, a higher early breast milk feeding proportion was associated with a higher discharge weight z-score in baseline-adjusted analyses. However, because infants in the low breast milk group were more premature, had lower birth weight, and required more intensive respiratory support, this association should not be interpreted as evidence of a direct causal effect. Rather, the observed association may largely reflect differences in prematurity, illness severity, and feeding readiness.

A higher early breast milk feeding proportion was independently associated with a higher discharge weight z-score in the primary multivariable model adjusted for gestational age, sex, and initial mechanical ventilation. This association showed an approximately linear pattern across the observed range of breast milk feeding proportion. However, the association was no longer statistically significant after additional adjustment for late-onset sepsis and necrotizing enterocolitis, suggesting that clinical complications may partially mediate or confound this relationship.

In our study, infants in the low breast milk group were more premature, had lower birth weight, and were more likely to require intensive respiratory support. This pattern likely reflects clinical and logistical challenges in the early postnatal period rather than a direct causal relationship. More immature and critically ill infants often experience delayed initiation and slower advancement of enteral feeding, increased reliance on parenteral nutrition, and difficulty in establishing adequate maternal milk supply.

In addition, maternal factors such as delayed lactogenesis, particularly following preterm delivery or maternal illness, may further limit early breast milk availability. As a result, infants with greater illness severity may receive a lower proportion of breast milk during the early postnatal period.

These baseline differences strongly suggest substantial confounding by prematurity and illness severity, and the observed association may largely reflect underlying clinical vulnerability rather than a direct causal relationship.

Our findings are consistent with a substantial body of evidence supporting the benefits of human milk feeding in preterm infants. Breastfeeding and human milk exposure have been associated with improved short- and long-term health outcomes, including reduced morbidity and mortality, through nutritional, immunologic, and anti-inflammatory mechanisms [1]. Current international guidelines emphasize human milk as the preferred source of enteral nutrition for preterm infants whenever possible [6]. However, prior studies examining the relationship between human milk feeding and growth have reported inconsistent findings. Colaizy et al. demonstrated that higher proportions of human milk intake were associated with reduced weight gain in very low birth weight infants, suggesting a potential trade-off between nutritional adequacy and the protective effects of human milk. Similarly, Brownell et al. reported a dose–response relationship in which increasing proportions of donor human milk were associated with decreased growth velocity.

These discrepancies may reflect differences in study design, including variations in fortification practices, timing and composition of enteral nutrition, and baseline characteristics of the study populations. In addition, many prior studies categorized feeding exposure into binary or ordinal groups, which may obscure potential dose–response relationships. In contrast, our study modeled breast milk feeding proportion as a continuous variable and applied restricted cubic spline analysis, allowing for a more detailed assessment of the exposure–outcome relationship.

Previous studies have demonstrated that early human milk feeding is associated with reduced risks of late-onset sepsis and necrotizing enterocolitis in very low birth weight infants [3,5]. These complications are well known to adversely affect nutritional tolerance, growth velocity, and overall clinical outcomes in the NICU [2,7]. The incidence of necrotizing enterocolitis in our cohort appears higher than that reported in many large international studies, including the Vermont Oxford Network. Notably, this difference cannot be readily explained by gestational age alone, as the average gestational age in our cohort was not lower than that of typical VLBW populations in these databases. Although factors such as differences in case ascertainment, institutional practices, and variability in diagnostic interpretation may have contributed to the observed incidence, these factors are unlikely to fully account for the magnitude of the difference. Therefore, the relatively high NEC rate in our cohort remains only partially explained and should be interpreted with caution. Importantly, because NEC is strongly associated with impaired growth, prolonged hospitalization, and mortality, it may act as an important confounder or mediator in the relationship between breast milk feeding and growth outcomes. This consideration further supports cautious interpretation of the observed associations.

Several factors may explain the higher NEC rate observed in our study. First, our cohort included a high-risk population of very preterm infants with lower gestational age, which is a well-established risk factor for NEC. Second, differences in diagnostic criteria, including the inclusion of clinically diagnosed cases in addition to confirmed cases, may have contributed to higher reported incidence. Third, as a tertiary referral center, our institution may have a higher proportion of critically ill infants, leading to potential referral bias. In addition, institutional variations in feeding practices and clinical management may also influence NEC incidence.

Similar considerations apply to late-onset sepsis. The incidence of late-onset sepsis may vary widely depending on patient characteristics, infection control practices, and diagnostic criteria across institutions. In high-risk populations such as very preterm infants, higher rates may be observed.

Importantly, NEC and late-onset sepsis are both strongly associated with impaired growth, prolonged hospitalization, and increased mortality. Therefore, these complications may act as important confounders or mediators in the relationship between breast milk feeding and growth outcomes. This may partly explain why the association was no longer statistically significant in the sensitivity analysis. The higher rates of late-onset sepsis and necrotizing enterocolitis observed in the low breast milk group may also reflect underlying prematurity and clinical vulnerability rather than a direct consequence of lower breast milk exposure.

Furthermore, early postnatal growth in preterm infants has been shown to influence subsequent growth and neurodevelopmental outcomes, underscoring the clinical relevance of optimizing early nutritional strategies [2]. Some studies have suggested slower early weight gain with higher proportions of human milk intake in preterm infants [13,14], whereas overall findings remain inconsistent across studies [15]. However, more recent studies and reviews suggest that with appropriate fortification and nutritional management, human milk–based feeding can support adequate growth while preserving its protective effects [4,12]. Our findings add to this literature by demonstrating a positive association between the proportion of breast milk intake and discharge weight z-score, rather than a simple comparison between feeding categories.

Unlike many previous studies that categorized feeding exposure into binary or ordinal groups, we modeled breast milk feeding proportion as a continuous variable and explored dose–response relationships using restricted cubic spline analysis. This approach avoids information loss associated with arbitrary categorization and allows more flexible modeling of exposure–outcome relationships [10,11]. The absence of clear nonlinearity in our spline analysis suggests that the association between early breast milk feeding proportion and discharge weight z-score is approximately linear within the observed range. The regression coefficients should be interpreted as reflecting local average associations within the observed distribution of breast milk exposure, and not as implying a linear effect across the entire 0–100% range. Beyond their high incidence and strong association with adverse outcomes, the loss of statistical significance after additional adjustment for late-onset sepsis and necrotizing enterocolitis raises important considerations regarding their roles as confounders or mediators. Infants with greater illness severity may be less likely to receive a higher proportion of breast milk and more likely to experience impaired growth, suggesting a potential confounding role. At the same time, early breast milk feeding may be associated with a lower risk of postnatal complications, which could in turn be linked to growth outcomes, suggesting a possible mediating pathway.

In our mediation analysis, a substantial portion of the association between breast milk feeding proportion and discharge weight z-score was mediated through late-onset sepsis, whereas necrotizing enterocolitis showed a more limited mediating role. These findings support the hypothesis that human milk may promote growth indirectly by reducing infectious morbidity rather than solely through direct nutritional effects. Importantly, these findings should not be interpreted as evidence of a definitive causal pathway, but rather as supporting a potential mechanism that warrants further investigation in prospective studies. However, these mediation findings should be interpreted with caution. Given the retrospective observational design, residual confounding and uncertainty in temporal relationships cannot be fully excluded. Therefore, the mediation analysis should be considered exploratory and hypothesis-generating rather than definitive evidence of causal mechanisms.

Together, these findings underscore the complex interplay between nutrition, illness severity, postnatal complications, and growth in preterm infants. Human milk feeding may influence growth not only through direct nutritional mechanisms but also indirectly by reducing complications that impair feeding tolerance and growth trajectories.

From a clinical perspective, our results support efforts to maximize breast milk exposure during the early postnatal period in preterm infants. The approximately linear association observed suggests that even incremental increases in breast milk feeding proportion may be associated with measurable growth benefits. The adjusted marginal effect analysis provides clinically interpretable estimates that may assist clinicians in counseling families and setting realistic nutritional goals in the NICU. These findings suggest that even partial increases in breast milk feeding proportion, rather than exclusive feeding, may provide measurable benefits in growth outcomes. This may support more flexible and individualized nutritional strategies in clinical practice, particularly in situations where full breast milk feeding is not feasible. In addition, considering the potential mediating role of postnatal complications, strategies aimed at increasing breast milk exposure may contribute not only to nutritional support but also to the reduction in morbidity in preterm infants.

The strengths of this study include the use of detailed enteral feeding records, standardized growth assessment using the Fenton growth reference [12], and robust statistical modeling incorporating both linear and non-linear approaches. The relatively large sample size and inclusion of consecutive NICU admissions further enhance the validity of our findings.

Several limitations should be acknowledged. First, the retrospective observational design precludes causal inference. Despite adjustment for multiple perinatal and clinical variables, residual confounding by prematurity, illness severity, feeding readiness, and clinician-driven treatment decisions cannot be excluded. Observational analyses cannot fully account for confounding related to clinical severity and treatment selection. In addition, a complete-case analysis was performed, and no imputation methods were applied. Although the proportion of missing data was small, this approach may introduce selection bias if missingness was not completely at random. An additional limitation is that mother’s own milk and donor human milk were analyzed together as a single exposure variable. These two sources differ in nutritional composition and bioactive properties, particularly because donor milk typically undergoes pasteurization, which may reduce certain immunological and enzymatic components. Therefore, the observed associations may not fully capture potential differences in growth effects between these milk sources. Future studies that distinguish between mother’s own milk and donor human milk are needed to better understand their differential effects on growth outcomes in preterm infants. Finally, longer-term growth and neurodevelopmental outcomes were not assessed.

## 5. Conclusions

In this retrospective cohort study of preterm infants, a higher proportion of breast milk feeding during the first 14 days of life was associated with a higher discharge weight z-score in the primary adjusted analysis. The relationship appeared approximately linear across the observed range. However, this association was no longer statistically significant after additional adjustment for major neonatal complications. Exploratory mediation analysis suggested that pathways involving late-onset sepsis may partly explain this association, whereas the mediating role of necrotizing enterocolitis appeared more limited. These findings should not be interpreted as evidence of a causal effect of breast milk proportion on growth outcomes, but rather as observational associations, given the substantial potential for residual confounding by prematurity and illness severity.

## Figures and Tables

**Table 1:** Baseline Characteristics of the Study Population According to Early Breast Milk Feeding Proportion.

Variable	High BM (n = 587)	Low BM (n = 587)	*p*-Value
GA (weeks)	29.66 ± 1.60	28.25 ± 1.89	<0.001
Male	324 (55.2)	314 (53.5)	0.598
Birth weight (g)	1219.17 ± 204.90	1092.26 ± 254.60	<0.001
Delivery mode, vaginal	220 (37.5)	227 (38.7)	0.7184
Multiple birth	125 (21.3)	151 (25.7)	0.085
Antenatal steroid use	372 (63.4)	375 (63.9)	0.903
IMV	283 (48.2)	339 (57.8)	0.001
Surfactant use	216 (36.8)	273 (46.5)	0.001
LOSEP	66 (11.2)	153 (26.1)	<0.001
NEC	102 (17.4)	110 (18.7)	0.595
LOS (days)	47.36 ± 19.37	60.83 ± 21.88	<0.001
Discharge weight (g)	2257.42 ± 379.63	2265.25 ± 384.64	0.651
BMP (%)	83.26 ± 4.24	71.40 ± 5.01	<0.001

Values are presented as mean ± standard deviation or frequency (percent). High and low breast milk (BM) groups were defined according to the median breast milk feeding proportion during the first 14 days of life. All *p*-values were obtained by independent two sample *t*-test or Mann–Whitney U test for quantitative variables, and chi-square test for qualitative variables. BMP, Breast milk proportion; GA, Gestational age; IMV, Initial Mechanical ventilation; LOS, Length of stay; LOSEP, Late-onset sepsis; NEC: Necrotizing enterocolitis.

**Table 2:** Univariable linear regression analyses for discharge weight z-score.

Variable	β (95% CI)	*p*-Value
BMP (%)	0.024 (0.016, 0.032)	<0.001
GA (weeks)	0.094 (0.060, 0.128)	<0.001
Birth weight (per 100 g)	0.218 (0.194, 0.242)	<0.001
Male sex (vs. female)	−0.171 (−0.286, −0.056)	0.004
Delivery mode (vaginal vs. cesarean)	0.128 (0.011, 0.244)	0.032
Multiple birth (yes)	−0.017 (−0.153, 0.119)	0.807
Antenatal steroid (yes)	−0.037 (−0.154, 0.080)	0.530
Surfactant use (yes)	−0.159 (−0.279, −0.039)	0.009
LOSEP (yes)	−0.737 (−0.893, −0.581)	<0.001
NEC (yes)	−1.025 (−1.176, −0.875)	<0.001

β represents the change in discharge weight z-score associated with each predictor in univariable linear regression models. For breast milk proportion, β coefficients represent the change per 1% increase; for interpretability, corresponding effect estimates are described in the text per 10% increase. BMP, Breast milk proportion; CI, Confidence interval; GA, Gestational age; LOSEP, Late-onset sepsis; NEC: Necrotizing enterocolitis.

**Table 3:** Multivariable linear regression analyses for discharge weight z-score, including primary and sensitivity models.

Variable	Primary Analysis Model		Sensitivity Analysis Model	
	β (95% CI)	*p*-Value	β (95% CI)	*p*-Value
BMP (%)	0.018 (0.009, 0.026)	<0.001	0.005 (−0.002, 0.013)	0.171
GA (weeks)	0.034 (−0.004, 0.072)	0.076	0.041 (0.007, 0.074)	0.018
Male	−0.165 (−0.277, −0.053)	0.004	−0.162 (−0.262, −0.062)	0.001
IMV	−0.286 (−0.401, −0.170)	<0.001	−0.212 (−0.316, −0.109)	<0.001
LOSEP	-	-	−0.617 (−0.757, −0.477)	<0.001
NEC	-	-	−0.976 (−1.114, −0.837)	<0.001

β coefficients represent the change in discharge weight z-score associated with a one-unit increase in each predictor. For breast milk proportion, β coefficients represent the change per 1% increase; for interpretability, corresponding effect estimates are described in the text per 10% increase. The primary analysis model was adjusted for gestational age, sex, and initial mechanical ventilation. The sensitivity analysis model was additionally adjusted for late-onset sepsis and necrotizing enterocolitis. BMP, Breast milk proportion; CI, Confidence interval; GA, Gestational age; IMV, Initial Mechanical ventilation; LOSEP, Late-onset sepsis; NEC: Necrotizing enterocolitis.

## Data Availability

The datasets generated and/or analyzed during the current study are available from the corresponding author on reasonable request, due to privacy and ethical restrictions.

## Abbreviations

The following abbreviations are used in this manuscript:

BMP	Breast milk proportion
CI	Confidence interval
EMR	Electronic medical record
GA	Gestational age
IMV	Initial mechanical ventilation
LOSEP	Late-onset sepsis
NEC	Necrotizing enterocolitis
NICU	Neonatal intensive care unit
